# Image-guided radiotherapy reduces the risk of under-dosing high-risk prostate cancer extra-capsular disease and improves biochemical control

**DOI:** 10.1186/s13014-018-0978-1

**Published:** 2018-04-12

**Authors:** Per Munck af Rosenschold, Michael J. Zelefsky, Aditya P. Apte, Andrew Jackson, Jung Hun Oh, Elliot Shulman, Neil Desai, Margie Hunt, Pirus Ghadjar, Ellen Yorke, Joseph O. Deasy

**Affiliations:** 1 Department of Medical Physics, Memorial Sloan Kettering Cancer Center, New York, USA; 2 Department of Radiation Oncology, Rigshospitalet, Copenhagen, Denmark; 3 Department of Radiation Oncology, Memorial Sloan Kettering Cancer Center, 1275 York Avenue, Box 22, New York, NY 10065 USA; 4 Department of Radiation Oncology, University of Texas Southwestern, Dallas, TX USA

**Keywords:** Image-guided, Radiotherapy, Prostate cancer, High risk disease, IMRT, Tumor control probability

## Abstract

**Background:**

To determine if reduced dose delivery uncertainty is associated with daily image-guidance (IG) and Prostate Specific Antigen Relapse Free Survival (PRFS) in intensity-modulated radiotherapy (IMRT) of high-risk prostate cancer (PCa).

**Methods:**

Planning data for consecutive PCa patients treated with IMRT (*n* = 67) and IG-IMRT (*n* = 35) was retrieved. Using computer simulations of setup errors, we estimated the patient-specific uncertainty in accumulated treatment dose distributions for the prostate and for posterolateral aspects of the gland that are at highest risk for extra-capsular disease. Multivariate Cox regression for PRFS considering Gleason score, T-stage, pre-treatment PSA, number of elevated clinical risk factors (T2c+, GS7+ and PSA10+), nomogram-predicted risk of extra-capsular disease (ECD), and dose metrics was performed.

**Results:**

For IMRT vs. IG-IMRT, plan dosimetry values were similar, but simulations revealed uncertainty in delivered dose external to the prostate was significantly different, due to positioning uncertainties. A patient-specific interaction term of the risk of ECD and risk of low dose to the ECD (*p* = 0.005), and the number of elevated clinical risk factors (*p* = 0.008), correlate with reduced PRFS.

**Conclusions:**

Improvements in PSA outcomes for high-risk PCa using IG-IMRT vs. IMRT without IG may be due to improved dosimetry for ECD.

**Electronic supplementary material:**

The online version of this article (10.1186/s13014-018-0978-1) contains supplementary material, which is available to authorized users.

## Background

Image Guided Radiation Therapy (IGRT) refers to the use of imaging to improve dose localization to targeted tissue regions [1]. Daily IGRT registered to fiducial markers in prostate cancer (PCa) radiotherapy allows geometric accuracy review and compensatory treatment couch shift. This reduces uncertainty of dose delivery, and is essential for highly conformal intensity modulated radiotherapy (IMRT).

Concurrent reduction in target volume margins to reduce normal tissue dose [1, 2] and facilitation of dose-escalation, which is associated with improved Prostate Specific Antigen (PSA) Relapse Free Survival (PRFS) [3–8], are previously theorized mechanisms whereby IGRT may improve the therapeutic ratio of IMRT. In the era of increasingly conformal RT, a number of publications have shown that the geometrical specifics of planning and delivery parameters may influence treatment outcome. Specifically, larger Rectum Cross Section (RCS) area at treatment simulation has been associated with inferior biochemical tumor control outcomes [9, 10]. In another cohort, where an IGRT protocol was used, no association between large RCS and worse PRFS was present [11]. As a caution, one study even found that the introduction of daily IGRT coupled with a reduced PTV margin was associated with an inferior PRFS [12].

At our institution, however, we initially introduced IG without altering target margins, dose, or planning directives and yet detected an improved PRFS for high-risk PCa [13]. Use of IG has been associated with reduced urinary and gastrointestinal-related toxicity [13–15].

High-risk prostate cancer is commonly associated with disease in the postero-lateral aspect of the gland, which comprises the peripheral zone and beyond it. The purpose of the current study was to quantitatively model variances in the dose delivery at areas of the prostate and in the area of extracapsular disease (ECD) in order to identify a potential mechanism for the benefit of IG-IMRT in PCa. We hypothesized that IGRT reduced statistical deviations from adequate irradiation that were occurring in the non-IG cohort, resulting in a reduced risk of under-dosing the posterolateral aspect of the prostate and at the site of likely ECD and improving disease control.

## Methods

### Patients and treatment protocol

We reviewed 102 NCCN (National Comprehensive Cancer Network v2008.2) high-risk prostate cancer patients consecutively treated at our institution during a transition to IGRT during 2005–2009, which represents the same high-risk cohort reported previously [13]. The patient characteristics are shown in Table 1 (Additional file 1: Table S1).

**Table 1:** Patient characteristics

Patient demographics	IG-IMRT (*n* = 35)		IMRT (*n* = 67)		*p*-value (Fisher)
	Number	Percent	Number	Percent	
Pre-treatment PSA (ng/ml)*					*p* = 0.169
< 10	15	42.9	41	62.1	
10–20	9	25.7	10	15.2	
> 20	11	31.4	15	22.7	
Total Gleason score*					*p* = 0.082
< 7	3	8.8	5	7.5	
7	10	29.4	8	11.9	
> 7	21	61.8	54	80.6	
T’stage*					*p* = 0.026
T1c-T2a	10	28.6	34	52.3	
T2b	9	25.7	6	9.2	
> T2b	16	45.7	25	38.5	
Radiation dose delivered					*p* = 1.000
86.4 Gy	34	97.1	66	98.5	
84.6 Gy	0	0.0	1	1.5	
82.. 8 Gy	1	2.9	0	0.0	
Age (y)					*p* = 0.071
< 70	12	34.3	37	55.2	
> 70	23	65.7	30	44.8	
Neoadjuvant ADT					p = 1.000
Yes	30	42.9	57	42.5	
No	5	7.1	10	7.5	
Positioning					p < 0.001
Prone	1	2.9	65	97.0	
Supine	34	97.1	2	3.0	
Number of risk factors**					*p* = 0.215
1	10	28.6	31	46.3	
2	18	51.4	27	40.3	
3	7	20.0	9	13.4	

*Abbreviations*: *PSA* Prostate Specific Antigen, *ADT* Androgen Deprivation

Therapy

*One, one and two of the patients lacked information on pre-treatment PSA, Gleason score and T stage, respectively

^**^Counting the number of risk factors as defined as PSA > 10, T Stage >T2c and total Gleason score > 7

Radiotherapy was planned in 1.8 Gy fractions to a total of 86.4 Gy using IMRT. The prostate, central part of the prostate (encompassing the urethra), rectum, bladder, bladder wall, were delineated. A planning target volume (PTV) margin of 1.0 cm except posteriorly where the margin was 0.6 cm was assigned to the clinical target volume (CTV), which encompassed the prostate plus seminal vesicles (see Additional file 2 additional details). Follow-up evaluations after IMRT were performed at 3 (1st year) to 6 months intervals. The median follow-up time was 3.0 (range: 0.3–4.8) and 4.9 (range: 0–7.4) years for the IG-IMRT and IMRT cohorts, respectively. PRFS was calculated using the Phoenix definition (nadir+ 2 ng/mL).

### Derivation of the risk of extra-capsular disease

**Fig. 1:**
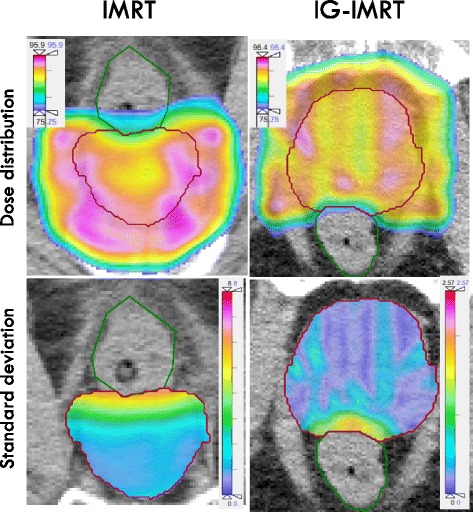
Transversal cross section (left) and sagittal plan (right) through the PTV of an example patient treated on the IMRT protocol with the bootstrap variance of the dose within the CTV shown in color-wash. It is apparent that the variance was larger within the CTV close to the rectum, which stems from a steep dose-gradient in that region as well as the magnitude of the positioning uncertainty in this direction

It is well known that positive margins in radical prostatectomy are associated with inferior PSA outcomes; hazard ratios of 1.2–3.7 have been reported. Further, a posterolateral positive surgical margin was observed to confer the greatest risk of recurrence [16]. This prompted us to study the dosimetry of the prostate capsule and beyond it in the peripheral zone, which in general is where the disease is located. For the purpose of this analysis, we defined 5 regions beyond the prostate gland. Each region concentrically isotropically expanded out 0.25 cm from the dorsal half of the CTV, which encompasses the posterolateral aspect of the prostate and the neurovascular bundle region. However, the regions were made such that they were not intersecting with the rectum (see Fig. 1, and Additional file 2 for a detailed description). Each patient’s T-stage, Gleason score and pre-treatment PSA were used to estimate the probability of ECD in the first region using a logistic regression model previously presented [17]. Two different data sets [18, 19] were identified that provides estimates of the risk of ECD as a function of distance out from the prostate gland (i.e. CTV). For this study, we used the average risk estimated from these two data sets to derive the risk of ECD in regions 2–5 (i.e. 0.25–1.00 cm out from the CTV).

### Simulation of treatments based on position uncertainty

See Additional file 2 for additional details on the procedure. Planned and delivered radiation dose distributions differ due to residual geometrical variations in setup, as well as other potential factors, such as anatomic changes during a course of radiotherapy. We attempted to quantify this difference for both the IMRT and IG-IMRT cohorts by means of simulating the positioning uncertainty, following a similar procedure as [20]. The patients’ planned dose distribution, CT data set and contours were extracted from the in-house treatment planning software and imported into the CERR software [21]. Using a programming script, the simulated isocenter position was sampled using a systematic and a random error using positioning uncertainty data from [22]. For IG-IMRT patients, however, the systematic error stems from lack of imaging isocentricity and misalignment vs. the radiation isocenter.

For both positioning protocols, 200 bootstrap samples (simulated treatments) for each patient were created. The list of positioning errors for each bootstrap were then used to shift the dose distribution and the total dose distribution was thus calculated by summation of the dose delivered for all the fractions. Also, the variance of the mean dose for each bootstrap sample for each patient was calculated for the PTV and CTV. Finally, using the bootstrap samples, the probability of a patient receiving a dose 10 Gy lower than the prescription dose to the posterolateral regions (defined above) was calculated.

### Statistical analysis

In this exploratory analysis, two-sided tests were used and unadjusted *p*-values of 0.05 or less were considered significant. Calculations were performed using R (v3.0.1). We investigated if RCS was associated with the dose to the posterior aspect of the PTV margin (the posterior quarter of the PTV defined from the cranio-caudal axis) (Spearman).

First, we were screened the covariates related to ECD for association with PRFS using univariate Cox regression. This pre-selection method was used to reduce the risk of over-fitting the ECD data, which were internally correlated. The ECD-related covariates included the patient-specific bootstrap risk of low dose to the posterolateral regions of the prostate gland (5 regions), and the risk of ECD in the same region, as well as the interaction parameter of the risk of ECD involvement and low dose (10 Gy or less lower than the prescription dose). Of these 15 covariates, the covariate with the strongest association with the PRFS in univariate analysis was referred to as the “candidate ECD descriptor”. Subsequently, the RCS, the bootstrap median dose to the CTV and the PTV, GS, T-stage and pre-treatment PSA, the number of clinical (NCCN) risk factors (i.e. GS 7+, PSA 10+ and T2c+) along with the candidate ECD descriptor were introduced in an univariate and multivariate Cox regression model for PRFS. The forward approach was used, stepwise introducing significant (*p* < 0.05) univariate parameters in the multivariate model (SPSS v19, IBM). The Cox model was generated in the same fashion for the whole cohort and subsequently for the IMRT cohort only. Kaplan-Meier plots were generated for the whole cohort (IG-IMRT and IMRT) and the IMRT cohort only, where patients were split into unfavourable and favourable groups based on the respective Cox models.

## Results

A comparison of dosimetric data for treatment plans and simulations (corrected for the actual number fractions delivered) for the IG-IMRT and IMRT cohorts is found in Table 2. The treatment plan average dose to the CTV was systematically higher, while the average dose to the posterior aspect of the PTV margin and the RCS were systematically lower and higher, respectively, for the IMRT cohort. For the whole cohort, increasing RCS was significantly associated with decreasing dose to the posterior aspect of the PTV (*p* < 0.001).

**Table 2:** Dosimetric data for each patient for the IG-IMRT and IMRT cohorts, respectively, extracted from the treatment plan (“PLAN”) and including the simulation of the positioning uncertainty (“SIMULATION”)

Parameter	IG-IMRT					IMRT					*p*-value (Mann-Whitney)
	Min	25% quartile	Median	75% quartile	Max	Min	25% quartile	Median	75% quartile	Max	
Rectal cross-section (cm2)	0.55	5.21	6.51	7.77	14.95	3.93	6.51	8.31	11.00	19.44	0.001
PLAN: Average dose to the CTV	83.29	87.10	88.17	88.52	89.80	85.58	87.77	88.54	89.22	91.24	0.008
PLAN: Average dose to the dorsal part of the PTV margin	79.48	80.73	83.36	84.20	86.77	76.43	79.89	81.46	83.33	87.03	0.005
SIMULATION: Expected dose to the whole CTV	83.25	87.03	88.12	88.46	89.70	84.26	86.51	87.24	88.26	90.07	0.039
SIMULATION: Variance of dose to whole CTV	0.24	0.29	0.34	0.38	0.74	1.55	2.16	2.48	2.88	3.89	0.000

Using the simulation approach, the median of the average dose to the CTV dropped less than 0.1 Gy and 1.3 Gy for the IG-IMRT and IMRT groups, respectively (*p* < 0.001). The ‘dose leakage’ out of the posterior part of the PTV margin was larger, 0.2 and 3.0 Gy for the IG-IMRT and IMRT cohorts, respectively. This dose leakage effect is shown graphically for a typical patient, where the variance of the dose is displayed in colour scale (Fig. 1). The expected ‘dose leakage’ out of the CTV was quite limited, even for IMRT. However, a subset of individual simulation cases had a very substantial dose leakage out of the prostate (i.e. more than 10% dose fall off) and these were more likely to have large simulated systematic positioning errors. For the IMRT cohort, a systematic positioning error that translated into an observed isocenter offset anteriorly was most strongly associated with increase dose leakage (Spearman rho = 0.44, *p* < 0.001), which is due to movement of the prostate into the sharp dose-gradient towards the rectum. In addition, but much less pronounced, an offset of the isocenter in the caudal direction was associated with a dose leakage (rho = 0.03, *p* < 0.001). A three-dimensional positioning error was strongly associated with dose leakage (rho = 0.38, *p* < 0.001), as well as the absolute deviation anterior-posteriorly (rho = 0.23, *p* < 0.001), laterally (rho = 0.03, *p* < 0.001) and cranio-caudally (rho = 0.31, *p* < 0.001).

**Fig. 2:**
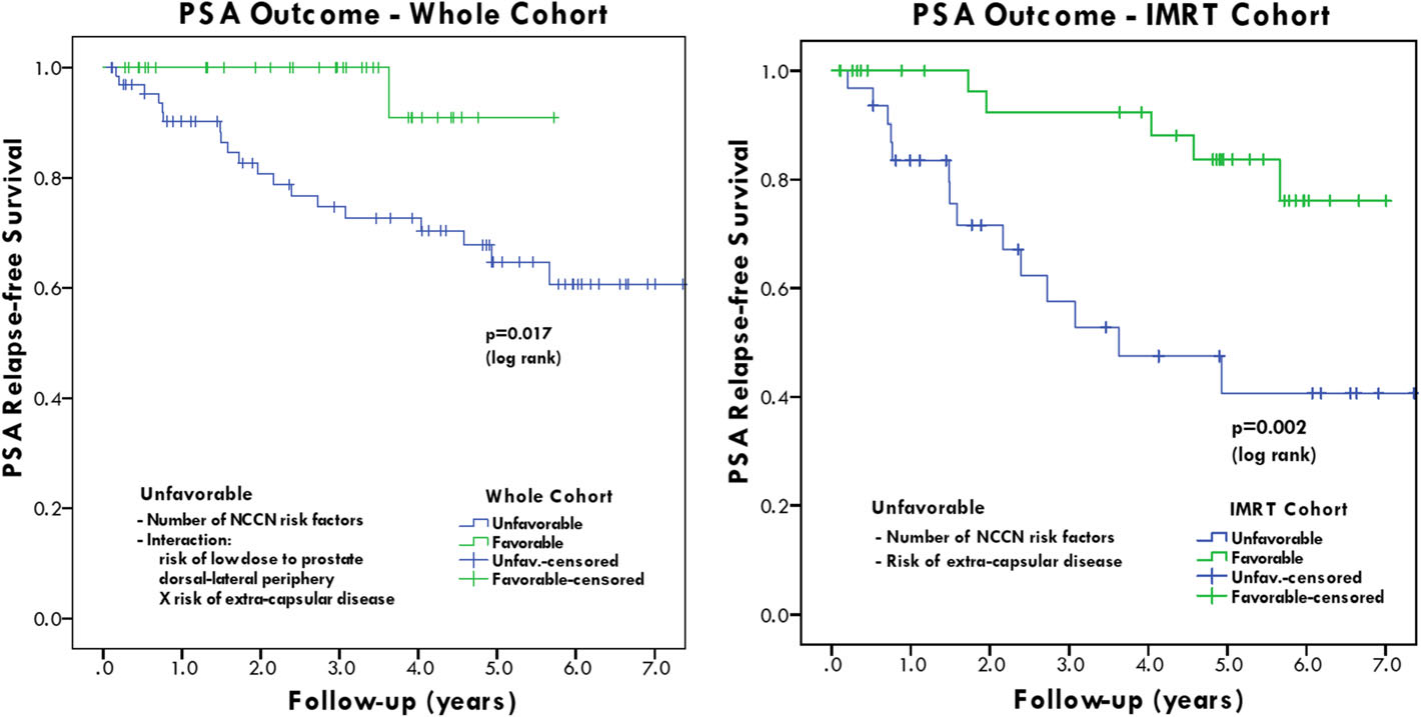
PSA relapse-free survival for high-risk prostate cancer patients for the whole cohort (left) and the IMRT cohort only (right). The respective Cox models were used to select the patients with favorable and unfavorable outcome, respectively

The interaction term of the probability of a 10 Gy dose lower than the prescription and the risk of ECD for the region at about 1 cm from the CTV was found be the ECD covariate with the strongest correlation with PRFS through univariate Cox regression (Additional file 1: Table S2). The multivariate Cox regression analysis demonstrated that the number of NCCN clinical risk factors and the interaction term of the probability of a 10 Gy dose lower than the prescription and the risk of ECD for the region at about 1 cm from the CTV was associated with a reduced PRFS (see Table 3, Additional file 1: Table S1 and Table S2). Increasing RCS was also significantly associated with a decreased dose to the posterior part of the PTV, but RCS alone was not associated with PSA failure for the combined group or for the IG-IMRT or IMRT groups individually. A Cox model for the *IMRT cohort only* is found in Additional file 1: Table S3, where the number of NCCN risk factors and the risk of ECD were significant in the multivariate analysis. Kaplan-Meier plots are found in Fig. 2 for the whole cohort (a) and for the (b) IMRT only cohort, respectively. In each of the plots, patients were split (median) into favourable and unfavourable groups based on the respective survival function estimated using the respective Cox model. Log rank statistics revealed significantly different PSA relapse free survival for favourable and unfavourable patients for the whole cohort (*p* = 0.017) and for the IMRT patients (*p* = 0.002).

**Table 3:** Univariate and Multivariate Cox model analysis data predicting for PRFS. Significant covariates in the univariate and multivariate model are printed in bold typeface

Cox model analysis Variables	Univariate analysis	Multivariate analysis		
	*p*-value	Hazard Ratio	95% CI	*p*-value
Overall model				0.001
T-Stage	0.083	ns		
Gleason	0.127	ns		
Pre-RT PSA	0.012	ns		
Number of NCCN risk factors^a^	0.008	2.15	(1.17–3.95)	0.013
Rectal Cross-section	0.558	ns		
Neo-adjuvant HT	0.395	ns		
Expected dose to the CTV	0.143	ns		
Candidate ECD descriptor^b^	0.005	3.88	(1.26–1 1.99)	0.0 18

^a^Pre-treatment PSA10+, G7+ and T2c+

^b^Interaction term: probability of low dose (10 Gy less than prescription dose) and probability of extra-capsular disease at about 1 cm dorso-laterally of the prostate gland

## Discussion

This is the first report to our knowledge to suggest that the observed improved PRFS with IG-IMRT could be related to the risk delivering a low dose to the dorsal region adjacent to the peripheral zone, which is the region known to be most likely harbouring disease. The derived hazard ratios related to ECD for our data are on par with those from data reported for radical prostatectomy series with positive margins [16]. Our finding emphasizes the significance of integration of IG radiation therapy treatment protocols for high-risk prostate cancer, where risk of disease progression and ECD are largest. The clinical decision was to keep the same PTV margins after the introduction of IGRT. Our analysis suggests that a reduction of the posterolateral PTV margins without target position correction could have reduced the PRFS of these high-risk patients as a direct result of target under-dosage. By comparison of the Cox model for the IMRT and the whole cohort, we find that the risk of low dose is the dose-volume factor that distinguishes the PSA outcome for IG-IMRT from IMRT patients. When inspecting the series of K-M curves in Fig. 2, we find that the use of IGRT appear to lift the unfavourable K-M curve up for the whole cohort towards the favourable group for IMRT only cohort. Our findings are supported by previous analyses, where the dose coverage near the prostate gland appear to have impacted the PSA outcomes for high risk prostate cancer patients [23, 24].

The positioning uncertainty data can be used to estimate the uncertainty of dose distributions for the IMRT and IG-IMRT cohorts by means of computer simulations. From these calculations we may observe statistical measures from the patient-specific probability distributions. We cannot, however, in retrospect identify the true positioning errors or the exact delivered dose distributions, only potential scenarios for the patients. An additional limitation of our study is that 3D–translations of the prostate gland were simulated, which may or may not reflect the motion experienced by the whole gland and the vesicles [25]. Further, we have disregarded intra-fraction motion, which may vary (marginally) for supine and prone positioning [26]. In addition, potential rotations or deformations were not included in the simulation model. It is however evident from this analysis the IMRT group tended to experience a larger systematic positioning errors, and this cohort of patients were consequently more likely to experience larger dosimetric deviations. In essence, if enough fractions are delivered without IG, large errors will eventually occur, which appears to have been compromising PSA outcomes. We show that, using the bootstrap samples of the patients, that the interaction between the risk of low dose to the posterolateral aspect of the prostate and the risk of ECD to said region is associated with PSA failure. Interestingly, also the number of clinical risk factors remained significant in the multivariate analysis, suggesting that a subset of the high-risk group have a larger risk of PSA failure regardless of IG use, possibly due to failure to obtain intra-prostatic local control. Failure to obtain local control for patients with multiple clinical risk factors is consistent with the data presented by Levegrun et al. [27], where having multiple risk factors was strongly associated to the probability of biopsy verified residual disease. Interestingly, limiting the analysis to only the IMRT cohort still yields a strong correlation the risk of ECD and with the number NCCN risk factors (Additional file 1: Table S3). Generally, the ECD related covariates show correlation with PRFS (Additional file 1: Table S2), though the covariates are also (unsurprisingly) internally correlated (data not shown). The selection of a 10 Gy dose drop is relatively arbitrary but was selected to represent a substantial dose reduction, likely to affect local control; however selecting 8 or 12 Gy produces similar results (data not shown). The multivariate Cox model of PRFS offers no proof in itself, but may offer a potential explanation of the observed difference between IG-IMRT and IMRT PSA outcomes and could be considered in the hypothesis generation for further studies. A full patterns of failure analysis might be helpful in that respect; this is however beyond the scope of the present work.

**Fig. 3:**
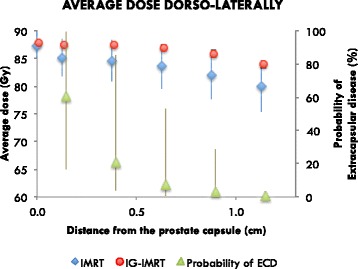
Dose fall-off in the posterolateral direction, averaged over all patients for all treatment plans. This is also averaged between the left and right regions for each patient. The 0 cm distance point is the average of the whole CTV. The probability of the existence of extra-capsular disease for the whole cohort of high-risk patients is plotted onto the secondary axis

The patient characteristics for the high-risk PCa IG-IMRT and IMRT groups selected for analysis were similar and only the T stages were significantly higher for the IG-IMRT group (Table 1). A single patient in the IG-IMRT group showed a markedly reduced lower delivered dose than the majority due to the exclusion of two fractions. One difference between the two cohorts was that IG-IMRT patients we treated in supine position while IMRT patients were mostly treated in prone position, which has probably influenced the positioning uncertainty somewhat [28]. Using the simulations of positioning uncertainty, we find that there were only minor differences with respect to the average dose to the CTV delivered to the IG-IMRT and IMRT cohorts, as shown in Fig. 3. The average dose posterolaterally is however substantially reduced for the IMRT cohort. This means that for most patients the delivered dose distribution inside the CTV will be close to the plan even without IGRT. However, without the use of IG there is a certain risk for a substantial reduction of the dose, which is practically non-existing for IG-IMRT patients.

In contrast to previous reports [9, 10] we found no correlation of RCS and the risk of PSA failure in this cohort of patients. However, the RCS data in our cohort were mostly below the 11 cm^2^ cut-off used previously [9]. Further, we are unable to confirm the observation that use of IG removes the association of RCS and PSA failure [11]. Consistent with this analysis, RCS was not a predictor for PSA failure when using IG [29]. We note that the finding in this exploratory retrospective analysis should be verified in a larger study, and that there is still a need for a mechanistic tumor control probability model including a more realistic method to account for non-uniform intra-prostate tumor burden. A potential approach to refine the current model is to use e.g. magnetic resonance imaging to attempt to contour the gross tumor [30] as well as any suspected extra-prostatic disease. However, for the current data set pre-treatment magnetic resonance scans were only available for a subset of patients.

## Conclusion

In this study we find that the potential for large, though infrequent, setup errors in fractional delivery have the potential to be a primary cause for the outcomes difference between IMRT and IG-IMRT. This strengthens the hypothesis of improved outcome by use of fiducial-based IGRT, and supports the notion that all radiotherapy treatments should be preferably accompanied with some form of adequate image guidance of the prostate position that would be sufficient to rule out significant setup errors.

## Additional file

Additional file 1: **Table S1.** The patient characteristics, for patients experiencing PSA relapse and control, respectively. Table S2. UNIVARIATE COX MODELS FOR THE CANDIDATE ECE DESCRIPTOR. The selected candidate with the strongest association with PSA relapse is marked (*). Table S3. Univariate and Multivariate Cox model analysis data predicting for PRFS for Non-IGRT patients ONLY. The multivariate model improved by the inclusion of the ECD descriptor (p=0.036). (ZIP 487 kb)

Additional file 2: Generating treatment bootstrap samples from random positioning uncertainty for IG-IMRT and IMRT treated patients. (PDF 462 kb)

## Abbreviations

CTV: Clinical target volume
ECD: Extra-capsular disease
GS: Gleason Stage
IG: Daily image-guidance
IGRT: Image Guided Radiation Therapy
IMRT: Intensity-modulated radiotherapy
KM: Kaplan-Meier
NCCN: National Comprehensive Cancer Network
PCa: High-risk prostate cancer
PRFS: Prostate Specific Antigen Relapse Free Survival
PSA: Prostate Specific Antigen
PTV: Planning target volume
RCS: Rectum Cross Section
