# Supplementary material for: Image-guided radiotherapy reduces the risk of under-dosing high-risk prostate cancer extra-capsular disease and improves biochemical control
Source: Radiat Oncol. 2018 Apr 12;13:64. doi: 10.1186/s13014-018-0978-1 (PMC5898030; doi:10.1186/s13014-018-0978-1)
Supplement: Supplementary file 1 — Table S1. The patient characteristics, for patients experiencing PSA relapse and control, respectively. Table S2. UNIVARIATE COX MODELS FOR THE CANDIDATE ECE DESCRIPTOR. The selected candidate with the strongest association with PSA relapse is marked (*). Table S3. Univariate and Multivariate Cox model analysis data predicting for PRFS for Non-IGRT patients ONLY. The multivariate model improved by the inclusion of the ECD descriptor (p=0.036). (ZIP 487 kb) [file 13014_2018_978_MOESM1_ESM.zip › Suppl Table 4.pdf]

Additional file 1: Table S1. The patient characteristics, for patients experiencing PSA relapse and control, respectively.

Patient demographics	**PSA relapse** *(n=20)* *n*	*%*	**PSA control** *(n=82)* *n*	*%*	
Pre-treatment PSA (ng/ml)					p=0.53
<10	9	*45.0*	47	*58.0*	
10-20	5	*25.0*	14	*17.3*	
>20	6	*30.0*	20	*24.7*	
Total Gleason score					p=0.39
<7	0	*0.0*	8	*9.9*	
7	3	*15.0*	15	*18.5*	
>7	17	*85.0*	58	*71.6*	
T stage					p=0.44
T1c-T2a	7	*35.0*	37	*46.3*	
T2b	2	*10.0*	13	*16.3*	
>T2b	11	*55.0*	30	*37.5*	
Age (y)					p=0.01
<70	15	*75.0*	34	*41.5*	
≥70	5	*25.0*	48	*58.5*	
Neoadjuvant ADT					p=0.49
Yes	16	*80.0*	71	*86.6*	
No	4	*20.0*	11	*13.4*	
Number of risk factors					p=0.03
1	5	*25.0*	36	*43.9*	
2	8	*40.0*	37	*45.1*	
3	7	*35.0*	9	*11.0*	
