# Supplementary material for: Image-guided radiotherapy reduces the risk of under-dosing high-risk prostate cancer extra-capsular disease and improves biochemical control
Source: Radiat Oncol. 2018 Apr 12;13:64. doi: 10.1186/s13014-018-0978-1 (PMC5898030; doi:10.1186/s13014-018-0978-1)
Supplement: Supplementary file 1 — Table S1. The patient characteristics, for patients experiencing PSA relapse and control, respectively. Table S2. UNIVARIATE COX MODELS FOR THE CANDIDATE ECE DESCRIPTOR. The selected candidate with the strongest association with PSA relapse is marked (*). Table S3. Univariate and Multivariate Cox model analysis data predicting for PRFS for Non-IGRT patients ONLY. The multivariate model improved by the inclusion of the ECD descriptor (p=0.036). (ZIP 487 kb) [file 13014_2018_978_MOESM1_ESM.zip › Suppl Table 5.pdf]

Additional file 1: Table S2. UNIVARIATE COX MODELS FOR THE CANDIDATE ECE DESCRIPTOR. The selected candidate with the strongest association with PSA relapse is marked (*)

**IMRT AND IG-IMRT PATIENTS**		**ONLY IMRT PATIENTS**	
Variable	*p-value*	*Variable*	*p-value*
P_ECD_R1	0.178	P_ECD_R1	0.059
**P_ECD_R2**	**0.009**	**P_ECD_R2**	**0.000**
**P_ECD_R3**	**0.007**	**P_ECD_R3 (*)**	**0.000**
**P_ECD_R4**	**0.032**	**P_ECD_R4**	**0.001**
**P_ECD_R5**	**0.033**	**P_ECD_R5**	**0.001**
P_LOW_R1	0.724	P_LOW_R1	0.126
P_LOW_R2	0.923	P_LOW_R2	0.135
P_LOW_R3	0.558	P_LOW_R3	0.264
P_LOW_R4	0.323	P_LOW_R4	0.230
P_LOW_R5	0.143	P_LOW_R5	0.197
P_ECD_R1*P_LOW_R1	0.618	P_ECD_R1*P_LOW_R1	0.527
P_ECD_R2*P_LOW_R2	0.086	P_ECD_R2*P_LOW_R2	0.609
**P_ECD_R3*P_LOW_R3**	**0.007**	P_ECD_R3*P_LOW_R3	0.083
**P_ECD_R4*P_LOW_R4**	**0.044**	P_ECD_R4*P_LOW_R4	0.168
**P_ECD_R5*P_LOW_R5 (*)**	**0.005**	**P_ECD_R5*P_LOW_R5**	**0.042**

Key:
P_ECE_R# = the probability of extra-capsular disease 0-0.25 cm (R1), 0.25-0.5 cm (R2), 0.5-0.75 cm (R3), 0.75-1.0 cm (R4) and 1.0-1.25 cm (R5), respectively.
P_LOW_R# = probability of low dose (10 Gy less than the prescription dose) dorso-laterally at 0-0.25 cm (R1), 0.25-0.5 cm (R2), 0.5-0.75 cm (R3), 0.75-1.0 cm (R4) and 1.0-1.25 cm (R5), respectively.
