# Supplementary material for: Image-guided radiotherapy reduces the risk of under-dosing high-risk prostate cancer extra-capsular disease and improves biochemical control
Source: Radiat Oncol. 2018 Apr 12;13:64. doi: 10.1186/s13014-018-0978-1 (PMC5898030; doi:10.1186/s13014-018-0978-1)
Supplement: Supplementary file 1 — Table S1. The patient characteristics, for patients experiencing PSA relapse and control, respectively. Table S2. UNIVARIATE COX MODELS FOR THE CANDIDATE ECE DESCRIPTOR. The selected candidate with the strongest association with PSA relapse is marked (*). Table S3. Univariate and Multivariate Cox model analysis data predicting for PRFS for Non-IGRT patients ONLY. The multivariate model improved by the inclusion of the ECD descriptor (p=0.036). (ZIP 487 kb) [file 13014_2018_978_MOESM1_ESM.zip › Suppl Table 6.pdf]

Additional file 1: Table S3. Univariate and Multivariate Cox model analysis data predicting for PRFS for Non-IGRT patients ONLY. The multivariate model improved by the inclusion of the ECD descriptor (p=0.036).

**Cox model analysis**	Univariate analysis	Multivariate analysis		
Variables	*p-value*	*Hazard Ratio*	*95% CI*	*p-value*
Overall model				***<0.001***
T-Stage	0.027	ns		
Gleason	0.342	ns		
**Pre-RT PSA**	*0.012*	*ns*		
Number of NCCN risk factors *	0.001	***2.27***	***(1.09-4.72)***	***0.028***
Rectal Cross-section	0.123	ns		
Neo-adjuvant HT	*0.633*	ns		
Expected dose to the CTV	0.068	*ns*		
Candidate ECD descriptor **	*<0.001*	***1.04***	***(1.00-1.08)***	***0.084***

* Pre-treatment PSA10+, G7+ and T2c+

** Probability of extra-capsular disease at about 0.5 cm dorso-laterally of the prostate gland.
