# Supplementary material for: Image-guided radiotherapy reduces the risk of under-dosing high-risk prostate cancer extra-capsular disease and improves biochemical control
Source: Radiat Oncol. 2018 Apr 12;13:64. doi: 10.1186/s13014-018-0978-1 (PMC5898030; doi:10.1186/s13014-018-0978-1)
Supplement: Supplementary file 2 — Generating treatment bootstrap samples from random positioning uncertainty for IG-IMRT and IMRT treated patients. (PDF 462 kb) [file 13014_2018_978_MOESM2_ESM.pdf]

Generating treatment bootstrap samples from random positioning uncertainty for IG-IMRT and IMRT treated patients

Radiotherapy planning and image guidance

Radiotherapy was planned in 1.8 Gy fractions to a total of 86.4 Gy using IMRT. The prostate, central part of the prostate (encompassing the urethra), rectum, bladder, bladder wall, were delineated. A planning target volume (PTV) margin of 1.0 cm except posteriorly where the margin was 0.6 cm was assigned to the clinical target volume (CTV), which encompassed the prostate plus seminal vesicles. Dose-volume limiting optimization objectives were assigned to the rectum and the bladder during the planning process. In addition, care was taken so that dose hot-spots did not overlap with the urethra. The PTV dose was within 95-107% of the prescription dose if feasible, with the low dose tending to be located in the prostate/rectum interface. Patients treated with daily IG were positioned supine, and were initially set up to skin marks. Thereafter, orthogonal kilovoltage 2D radiographs (OBI, Varian Medical Systems, Palo Alto) were obtained, and the patients position were corrected using 3D translations if the deviation of fiducial marker positions exceeded 2 mm in any direction as compared to the position on the planning CT. A set of verification radiographs was afterwards taken to ensure that the new position was within 2 mm in all directions. The standard procedure was then to treat the patient without post-fraction imaging. Patients treated without daily IG were typically set up in prone position using skin marks, and the position was verified weekly using megavoltage images matched onto the bony anatomy of the planning CT.

Method for positioning used at MSKCC

The IMRT group (treated without IGRT) were typically in prone position (64 out of 67). Patients were positioned towards bony structures once a week, and the correction vector from skin marks to bony structures was recorded and used throughout the rest of the week. This procedure was repeated for the course of 45-48 fractions.

The patients treated with daily IGRT and IMRT (IG-IMRT) were first positioned using skin marks and this position was subsequently corrected using orthogonal x-rays daily and fiducial markers inside the prostate. The tolerance for the IGRT procedure was 2 mm (in each cardinal direction).

Method for sampling positioning variation

The position (P) will be sampled in the following way:

$$\left(P_i^{dir}\right)_x = \left(SE^{dir}\right)_x + \left(RE_i^{dir}\right)_x$$

Where *x* is sample number (1-200), and *dir* is direction (AP, CC, LR) and *i* is fraction number (typically 1-48). Note that SE is the systematic error and drawn once for each sample and RE is the random error. SE and RE are derived by

$$\left(SE^{dir}\right)_x = \left(SE^{IG}\right)_x + \left(SE_P^{dir}\right)_x$$

Where, index IG is the inherent uncertainty introduced by the accuracy of the imaging system and drawn once for each sample; 0.75 mm (1SD) in each direction. For IG-IMRT patients, the term indexed P is zero, and for or IMRT patients, then this is picked randomly from the distribution (assumed normal distribution) and from the data below. $\left(RE_i^{dir}\right)_x$ equals 1.4 mm (1SD) for patients treated with daily IGRT, stemming from the matching procedure (est. to be 1 mm) and the residual error tolerated of 2 mm (est. to equal to 2 SD). For IMRT patients the $\left(RE_i^{dir}\right)_x$ varies with direction, and were estimated using the data previously presented by our group (Munck af Rosenschold et al, 2014).

We split patients into four risk groups in terms of positioning uncertainty (Group 1-4, below) using BV (bladder volume at CT simulation) and RCS (rectum cross section area at CT simulation). Then, we pick randomly a patient number (see below) within each patient group for each bootstrap sample (1-200). Obviously, we might then pick the same series of SE and RE for a subsequent bootstrap sample. However, given that the SE and the RE are used distributions used for subsequent sampling, the same series of positioning deviations will not be reproduced in multiple bootstrap samples. The advantage of the method is that it retains the internal correlation of uncertainty estimates in all directions (cf. Munck af Rosenschold et al, Radiother Onc 2014). SE and RE for the patient groups are found in tables below.

Definition of dorsolateral region

For the purpose of this analysis, we defined 5 regions beyond the prostate gland. Each region concentrically were isotropically expanded out 0.25 cm from the dorsal half of the CTV (prostate). Thus, the regions encompass the posterolateral aspect of the gland including the neurovascular bundles. However, the regions were made such that they were not intersecting with the rectum (see schematic below).

Schematic of definition of dorsolateral regions for simulations (not to scale)

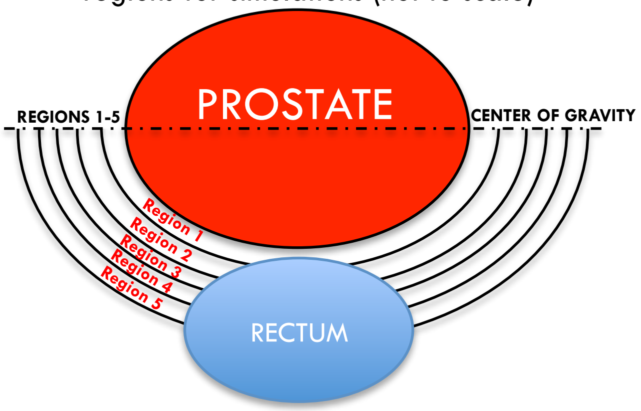

Dosimetric calculations

We attempted to quantify the radiation therapy dosimetry difference for the IMRT and IG-IMRT cohorts by means of simulating the positioning uncertainty, following a similar procedure as Zhang et al (Phys Med Biol 2006). The patients' planned dose distribution, CT

data set and contours were extracted from the in-house treatment planning software and imported into the CERR software (Deasy et al, Med Phys 2003). Using a programming script, the simulated isocenter position was sampled using a systematic and a random error. The sum of the dose is derived for each patient, each bootstrap sample and for each position in three-dimensional space:

$$[D_{tot}]_x = \left(\sum_{i}^{n} d_i\right)_x$$

Dtot is the sum dose matrix of a series of doses (*di*) from fractions (*n*), for bootstrap sample *x* (200 samples). From the *Dtot* dose matrix, we calculate the average and the standard deviation of the dose based on the fraction variations for the prostate. Finally, using the 200 bootstrap samples for each patient, the risk of receiving a dose reduction of at least 10 Gy lower than the prescription dose, in each of the Regions 1-5, is derived.

Positioning data

Systematic deviation (SE) and random deviations (RE) (1 sd) in each direction given in **cm** below.

**Group 1**	BV<204.2	RCS<6.148				
Number	SE_CC	RE_CC	SE_AP	RE_AP	SE_SD	RE_SD
1	-0.0187	0.2039	-0.0979	0.3889	-0.2000	0.3837
2	-0.3043	0.3782	-0.9851	0.3520	0.2426	0.4432
3	-0.1167	0.2652	-0.1125	0.3311	0.2292	0.3494
4	-0.0896	0.3737	-0.3812	0.3612	-0.2875	0.2590
5	0.2133	0.4299	-0.1356	0.4012	0.3956	0.2440
6	0.1167	0.3970	0.1896	0.3872	0.0500	0.2406
7	0.3146	0.3003	-0.2000	0.6507	0.2188	0.3456
8	-0.0089	0.2304	0.8978	0.4957	0.3067	0.3786
9	0.3292	0.2052	-0.2792	0.4068	0.0062	0.3076
10	0.2958	0.2705	0.9813	0.8763	-0.3292	0.2560
11	-0.1563	0.4272	-0.5646	0.5017	-0.2646	0.2149
12	0.3600	0.3003	-0.4600	0.4131	0.2556	0.2252
13	0.0708	0.2231	-0.1583	0.4833	-0.1729	0.2210
14	0.3447	0.2780	0.2957	0.6718	0.2191	0.1907
15	0.2208	0.2895	-0.1083	0.3274	-0.2375	0.2710
16	-0.3271	0.2773	-0.5250	0.5995	-0.0521	0.2658
17	0.1229	0.2363	0.2917	0.2360	0.3563	0.2865
18	0.0000	0.2393	-0.6644	0.3967	0.1667	0.4472
19	0.7933	0.3658	-0.4356	0.3638	0.1711	0.2361
20	1.0250	0.3901	-0.2205	0.5312	-0.1932	0.1946
21	0.0896	0.4874	-0.2979	0.3687	0.0417	0.2632
22	0.0468	0.2789	0.0404	0.3651	-0.3340	0.4350
23	0.1792	0.3128	0.3255	0.5716	0.0021	0.2497
24	0.3043	0.1978	-0.2106	0.5138	0.2596	0.3386
25	-0.2833	0.4081	-0.6896	0.5041	0.3083	0.2483
26	0.0250	0.2139	-0.1187	0.4541	-0.0604	0.2742
27	0.5489	0.3012	-0.1867	0.3666	0.1889	0.1555
28	0.3809	0.2482	-0.0553	0.3105	-0.2553	0.3289
29	0.6213	0.3587	-1.0447	0.3972	0.2447	0.2933
30	-0.1854	0.2609	0.1708	0.3128	-0.0229	0.1547
31	-0.1063	0.2846	-0.5521	0.4908	0.5375	0.2671
32	-0.1813	0.2750	0.2292	0.3914	0.2396	0.3547
33	0.0354	0.2597	-0.6813	0.4666	0.4563	0.2567
34	-0.4979	0.3380	-0.1667	0.2992	0.2438	0.1749
35	-0.1188	0.2170	0.2833	0.4023	0.2604	0.2688
36	0.2667	0.3587	0.4292	0.4410	-0.1167	0.2838
37	0.6386	0.3119	0.2409	0.4315	-0.0705	0.2620
38	0.1208	0.2361	0.0813	0.3133	0.0063	0.3454
39	-0.1938	0.2057	-0.1521	0.3679	0.2625	0.2695
40	0.1809	0.2473	0.0234	0.3038	0.0936	0.3371

41	0.4133	0.2625	0.3822	0.3985	0.1911	0.3260
42	-0.0146	0.2467	0.3292	0.3859	0.2000	0.2713
43	0.4830	0.4172	-0.7851	0.6175	0.0404	0.4781
44	0.2563	0.2736	-1.0396	0.4748	0.2958	0.2551
45	-0.0750	0.2302	-0.2354	0.4857	-0.5062	0.2794
46	-0.4042	0.2895	0.1771	0.3416	0.3062	0.2693
47	-0.0979	0.2026	-0.2146	0.4182	-0.0854	0.3003
48	0.1771	0.1882	0.3583	0.3758	0.1250	0.2840
49	0.3556	0.3829	0.4489	0.2974	0.3622	0.2259
50	-0.1479	0.2576	1.0375	0.4456	0.1667	0.3144
51	-0.0689	0.2601	0.0156	0.3943	-0.3044	0.2899
52	0.2771	0.2962	-0.0521	0.5295	-0.0083	0.3134
53	0.5067	0.2553	-0.6511	0.4049	0.0867	0.3721
54	0.4792	0.3332	0.0354	0.4484	0.1208	0.3215
55	0.2681	0.2890	-0.1723	0.4871	0.1787	0.2805
56	-0.0125	0.2090	0.0312	0.5149	0.5208	0.2982
57	0.3188	0.2623	-0.3771	0.4244	0.0375	0.1841
58	0.0229	0.2354	0.3458	0.3876	0.0771	0.3061
59	0.2229	0.3347	-0.8813	0.4276	0.3854	0.2895
60	-0.1792	0.1879	0.2750	0.2928	0.2354	0.2226
61	-0.5563	0.2728	0.0521	0.3345	-0.1333	0.3828
62	0.0000	0.3261	0.1844	0.3191	0.1911	0.2687
63	0.6362	0.2279	-0.4787	0.4043	-0.0447	0.2448
64	0.2356	0.3399	0.0422	0.6545	0.0711	0.3280
65	-0.0295	0.2278	0.0182	0.3931	0.2227	0.2786
66	0.1689	0.2255	-0.1489	0.3341	0.2667	0.3148
67	-0.1574	0.3412	-0.7021	0.6134	0.7404	0.4514
68	0.5271	0.2171	-0.4854	0.3843	0.0313	0.1323
69	0.3021	0.2605	0.4104	0.4204	-0.1250	0.1816
70	0.5188	0.2582	0.6562	0.3045	-0.0021	0.2779
71	-0.0208	0.2996	0.0021	0.3212	-0.1312	0.1870
72	-0.1563	0.2517	-0.2042	0.2902	-0.5625	0.3343
73	-0.0958	0.2333	-1.1333	0.4848	0.3812	0.1853

Group 2 BV>204.2 RCS<6.148

Number	SE_CC	RE_CC	SE_AP	RE_AP	SE_SD	RE_SD
1	-0.4667	0.4540	-0.2500	0.4860	-0.0542	0.2828
2	0.0617	0.2567	0.3447	0.3804	0.2000	0.2493
3	-0.1021	0.3442	-1.1063	0.4314	-0.1000	0.3747
4	0.1229	0.2452	-0.2750	0.3739	-0.0188	0.2455
5	0.0479	0.3501	0.4229	0.2762	-0.3833	0.4023
6	0.0511	0.2528	-0.5333	0.3960	0.0711	0.2170
7	0.2067	0.2725	-0.3156	0.4537	0.2067	0.2675
8	-0.2104	0.3429	0.2104	0.3959	0.1354	0.2320

9	0.0222	0.2430	-0.1533	0.4325	-0.2067	0.2416
10	0.5333	0.4597	-0.8000	0.4090	0.1911	0.2285
11	0.0125	0.6006	-0.2437	0.6565	0.1437	0.4141
12	0.3375	0.2557	0.0542	0.4156	-0.2062	0.2235
13	0.1000	0.2774	-1.0064	0.5387	0.2340	0.4420
14	0.3625	0.3779	-0.2458	0.3452	0.1958	0.2843
15	-0.2533	0.3355	0.0867	0.3865	0.0178	0.2774
16	-0.4667	0.4364	-0.1111	0.3511	0.2578	0.2360
17	-0.0792	0.2568	0.7313	0.3621	0.0729	0.3227
18	0.3583	0.2404	0.0646	0.2935	0.0188	0.2150
19	0.1711	0.2959	-0.1422	0.3408	0.2089	0.2043
20	0.2979	0.2986	0.3208	0.3897	0.0813	0.2687
21	-0.2167	0.2704	0.1857	0.5164	0.2786	0.2504
22	0.4604	0.3553	-0.7042	0.3281	0.0687	0.2882
23	-0.0042	0.3707	0.7000	0.4767	0.0479	0.4448
24	0.4083	0.2766	-0.2542	0.3741	0.0958	0.2767
25	0.0311	0.2334	-0.1533	0.4429	-0.0600	0.2649
26	0.1688	0.2594	-0.2312	0.3116	-0.1104	0.2034
27	0.3792	0.3307	0.5042	0.5971	0.2708	0.2052
28	0.2354	0.2855	-0.4625	0.3565	0.2000	0.2278
29	0.1854	0.2432	-0.0458	0.4771	-0.1687	0.2242
30	0.1854	0.2032	-0.1833	0.3417	0.0938	0.2530
31	-0.0733	0.2240	-0.0222	0.3140	0.0133	0.3314
32	0.1229	0.4430	0.5750	0.4866	-0.0583	0.4815
33	0.7771	0.3771	-1.1938	0.4931	-0.0729	0.4160
34	0.0109	0.2575	0.6326	0.5793	0.2870	0.2833
35	0.4333	0.7783	-0.3104	0.5586	0.0021	0.2855
36	-0.0800	0.1375	-0.1400	0.5136	-0.0378	0.2405
37	0.2267	0.2934	0.4822	0.4024	0.1978	0.2039
38	0.2813	0.3431	-0.1563	0.5709	0.2750	0.2436
39	0.5860	0.3502	-0.4395	0.4141	-0.0721	0.1667
40	-0.1979	0.2505	-0.1063	0.3316	0.0187	0.2331
41	0.2511	0.2997	0.1778	0.4073	-0.0933	0.2071
42	0.2600	0.3353	-0.0467	0.4208	-0.0800	0.2982
43	0.3667	0.4039	-0.9271	0.3746	-0.2021	0.4003
44	0.1044	0.4226	-0.4044	0.9802	-0.0956	0.1977
45	0.0083	0.2030	-0.2625	0.3207	0.2792	0.1713
46	0.6604	0.4036	-0.6083	0.4282	-0.2187	0.2472
47	-0.0311	0.2745	0.4156	0.4248	0.4244	0.2885
48	0.2688	0.2969	0.4187	0.3950	0.3021	0.3000
49	0.4000	0.2836	-0.5750	0.7009	-0.1875	0.2780
50	-0.2646	0.3710	0.5750	0.5916	0.2625	0.3717
51	-0.3533	0.2889	-0.0400	0.3658	0.0622	0.2480
52	0.0468	0.3161	-0.0830	0.4997	-0.4404	0.2998
53	0.2222	0.3343	-0.0022	0.3265	0.1111	0.1668
54	0.5087	0.3577	-0.3217	0.5711	0.0870	0.2680

55	-0.1042	0.2790	-0.1229	0.3089	0.1979	0.3070
56	0.1234	0.3840	-0.6617	0.3865	0.1574	0.3098
57	0.1313	0.2494	0.2250	0.3265	0.2958	0.1688
58	-0.0778	0.3029	-0.2511	0.3923	0.1044	0.1999
59	0.2417	0.2483	0.1604	0.5005	0.3833	0.4591
60	0.2646	0.2693	-0.1021	0.3773	0.1104	0.1992
61	-0.0542	0.1487	0.1396	0.3456	-0.0104	0.1601
62	0.1958	0.3294	-0.1979	0.4879	0.0125	0.4266
63	0.3354	0.2445	-0.4000	0.2881	-0.3708	0.3339
64	0.0208	0.2917	-0.6021	0.3623	-0.1750	0.3845
65	-0.4458	0.3445	-0.7833	0.5643	0.0146	0.2806
66	-0.0104	0.3520	-0.0958	0.3930	-0.1021	0.4629
67	-0.0978	0.2221	-0.1422	0.3823	0.2356	0.2621
68	0.6455	0.2327	0.1818	0.3768	-0.3068	0.2327

**Group 3**	BV<204.2	RCS>6.148				
Number	SE_CC	RE_CC	SE_AP	RE_AP	SE_SD	RE_SD
1	0.3378	0.2782	-0.4911	0.3118	0.0956	0.3303
2	0.4542	0.4202	-0.8437	0.4141	0.6333	0.2876
3	-0.1333	0.2836	0.4667	0.3649	0.4356	0.3990
4	-0.0333	0.2409	0.0771	0.3068	0.1542	0.3707
5	0.0000	0.3555	-0.5771	0.4214	-0.0063	0.2158
6	0.6667	0.3640	0.0292	0.3555	-0.0313	0.2860
7	0.0167	0.2898	0.0396	0.3023	-0.0208	0.2183
8	0.1978	0.2856	-0.1200	0.2573	-0.3956	0.3176
9	0.1188	0.2848	0.1417	0.4062	-0.2042	0.2736
10	0.4875	0.3387	-0.9313	0.3748	0.1958	0.2713
11	-0.0271	0.2171	0.1333	0.3899	0.4354	0.2497
12	0.0386	0.1883	-0.1045	0.4198	-0.0500	0.3317
13	-0.0667	0.4474	0.1396	0.4286	0.1625	0.3606
14	0.1708	0.2458	0.0458	0.3162	0.0479	0.2202
15	0.2646	0.3367	-0.6146	0.3941	0.0292	0.3870
16	-0.0156	0.2696	0.0733	0.4835	0.1533	0.3578
17	-0.0911	0.2275	-0.2711	0.4315	0.4689	0.2592
18	-0.2458	0.2535	0.1333	0.3905	0.1792	0.1989
19	0.4091	0.3529	-0.5682	0.4684	-0.0932	0.2823
20	0.1417	0.2559	-0.0438	0.3886	-0.1333	0.2816
21	0.2133	0.5260	-0.3267	0.8108	-0.0222	0.2285
22	-0.6822	0.2987	1.5467	0.5550	0.0511	0.2967
23	-0.2289	0.2096	-0.3311	0.4582	-0.0489	0.2997
24	0.2542	0.2501	-0.5417	0.3792	-0.2896	0.2399
25	-0.0875	0.3722	-0.1958	0.4125	0.2896	0.2595
26	0.0340	0.3824	0.3149	0.6362	-0.2894	0.3509
27	0.0222	0.2420	-0.4356	0.3445	0.4222	0.3295
28	0.3396	0.2541	-0.6458	0.3531	0.8583	0.3072

29	-0.6565	0.5286	0.9761	0.4143	-0.1891	0.4610
30	0.2733	0.2903	-0.8689	0.3377	-0.0644	0.3569
31	0.0911	0.2712	0.3800	0.5513	-0.0556	0.2073
32	0.2689	0.2819	-0.7778	0.4646	0.2489	0.3368
33	0.0844	0.3637	0.6133	0.5409	0.0222	0.2721
34	-0.2542	0.4146	0.6500	0.3281	0.5583	0.3017
35	-0.2896	0.2126	0.0417	0.4907	-0.1104	0.2897
36	0.4063	0.3698	-0.4458	0.4589	0.1208	0.4182
37	-0.2396	0.2549	-0.4000	0.3162	0.2833	0.2167
38	-0.3125	0.2438	0.5729	0.3847	0.3458	0.2736
39	-0.1489	0.3280	0.8578	0.4831	-0.1044	0.3275
40	-0.3771	0.2595	-0.1625	0.4988	0.0333	0.3090
41	-0.0378	0.3114	-0.5844	0.4959	-0.2378	0.3892
42	-0.1729	0.2796	-0.0792	1.0657	0.1188	0.4666
43	0.5208	0.4571	0.1915	0.3933	0.4750	0.2964
44	0.0911	0.2937	-0.2689	0.4425	0.0400	0.2562
45	-0.2833	0.3889	0.9104	0.6672	0.2312	0.3082
46	0.3044	0.2984	0.1089	0.6413	0.2933	0.2973
47	-0.3313	0.3656	-0.3229	0.3372	0.2542	0.2851
48	-0.1479	0.2752	0.6354	0.4300	0.1313	0.3321
49	0.1208	0.2568	0.4146	0.4042	0.0938	0.2273
50	0.2000	0.3122	-0.5208	0.3690	0.0771	0.2512
51	-0.2104	0.3075	-0.1375	0.4014	0.0375	0.2757
52	-0.0292	0.3059	-0.4750	0.8687	0.1833	0.5212
53	0.3083	0.3512	-0.6792	0.4277	-0.2896	0.4106
54	0.3979	0.4235	-0.1021	0.3310	0.2042	0.2673
55	-0.2708	0.3080	0.0208	0.4658	-0.3500	0.3519
56	0.0750	0.2654	-0.3500	0.4238	-0.1333	0.4614
57	0.1625	0.3571	-0.6833	0.5200	-0.1896	0.3610
58	0.2396	0.3120	-0.0875	0.5413	0.2250	0.3098
59	0.0646	0.2016	-1.1063	0.5393	-0.2750	0.2810
60	0.1467	0.2897	-0.1341	0.4670	0.2933	0.3319
61	0.0021	0.3252	-0.2250	0.3889	0.0083	0.2201
62	-0.2104	0.1848	0.7646	0.3693	0.1521	0.2790
63	0.0467	0.4049	-0.3156	0.5304	0.4000	0.3053

**Group 4**	BV>204.2	RCS>6.148				
Number	SE_CC	RE_CC	SE_AP	RE_AP	SE_SD	RE_SD
1	-0.1958	0.3476	-0.5729	0.3940	0.4083	0.3059
2	-0.0813	0.2515	0.9104	0.3191	-0.0250	0.2654
3	0.0917	0.2465	-0.5125	0.2922	0.3187	0.2170
4	-0.2083	0.3488	-0.2771	0.3771	-0.0125	0.2247
5	0.5489	0.3079	0.1867	0.4383	-0.1933	0.2692
6	-0.1646	0.3588	-0.2021	0.6809	-0.0667	0.3205

7	0.2438	0.3798	-0.2938	0.4479	0.2125	0.2170
8	-0.1521	0.2851	-1.4708	0.3142	0.5187	0.1875
9	-0.0375	0.2376	0.6167	0.4478	0.3521	0.2010
10	-0.0583	0.2835	0.3938	0.2374	0.1104	0.2746
11	0.2489	0.2096	-0.0889	0.4458	0.1089	0.2485
12	0.1313	0.9214	0.2083	0.4694	-0.0333	0.2999
13	0.4729	0.3752	-0.5292	0.4594	0.3396	0.3344
14	-0.2652	0.3749	1.5087	0.5253	0.1500	0.2934
15	-0.0178	0.2026	-0.1489	0.3195	-0.1200	0.2616
16	-0.4688	0.2519	0.6604	0.3292	0.0167	0.5799
17	0.2133	0.6642	-0.3356	0.5068	0.0267	0.3236
18	0.1109	0.4443	-0.1696	0.4273	-0.0239	0.3288
19	0.1188	0.3272	0.8229	0.4926	0.3021	0.3949
20	0.2333	0.4526	-0.3458	0.3775	0.3833	0.2373
21	0.3333	0.3097	0.1133	0.4832	0.1644	0.2656
22	-0.1917	0.3188	-0.1188	0.5221	-0.2042	0.3242
23	-0.7729	0.4186	0.7729	1.2576	0.0396	0.4471
24	-0.6396	0.2789	-0.7229	0.4922	-0.2063	0.2740
25	-0.2063	0.3111	-0.3271	0.4685	0.2938	0.2629
26	0.4604	0.2719	-0.2958	0.4496	0.2354	0.1995
27	0.1438	0.2287	0.6896	0.3544	-0.1333	0.3110
28	0.5938	0.5829	-0.7917	0.7136	-0.2188	0.2893
29	0.3295	0.2775	-0.1750	0.3404	-0.0045	0.3510
30	0.1604	0.2908	-0.3271	0.3972	-0.2146	0.2297
31	0.1500	0.2917	-0.5312	0.2918	0.4521	0.2903
32	0.3417	0.3228	-0.1917	0.5193	0.0021	0.2058
33	0.3889	0.2773	0.6978	0.4309	0.3644	0.2846
34	0.0271	0.1954	-0.3271	0.3147	0.1354	0.2914
35	-0.1458	0.2432	0.4313	0.4043	-0.2125	0.3577
36	0.5000	0.3307	-0.6609	0.8280	0.0604	0.2295
37	0.1625	0.3133	1.1792	0.8508	0.1187	0.2871
38	-0.0400	0.3683	0.0756	0.3797	0.0089	0.3232
39	0.2583	0.5910	-0.5062	1.0022	-0.2083	0.4292
40	-0.1771	0.2611	0.3667	0.3478	-0.0729	0.2574
41	-0.0391	0.4404	-0.0348	0.4463	-0.4087	0.4386
42	-0.1067	0.3041	-0.8511	0.4088	0.0489	0.2191
43	0.0644	0.2248	-0.7558	0.5624	-0.1602	0.2985
44	0.3667	0.3341	0.8792	0.4376	0.2250	0.2862
45	-0.1111	0.1898	0.4311	0.4461	0.5000	0.3148
46	0.0851	0.2897	-0.1894	0.5665	0.2064	0.2399
47	0.0000	0.2895	-0.7792	0.3038	-0.0604	0.3133
48	0.1396	0.3356	-0.0875	0.4975	0.2542	0.3591
49	-0.2979	0.3864	1.3043	0.5793	0.1532	0.3966
50	0.0813	0.4286	-0.2375	0.5484	0.0687	0.3904
51	0.1479	0.3268	-0.8208	0.4749	-0.2667	0.4334
52	0.3400	0.2597	0.0889	0.3393	0.1311	0.2193

53	-0.0354	0.3111	0.2813	0.2929	0.3208	0.2526
54	0.1146	0.4736	0.1146	0.3679	-0.1042	0.2414
55	-0.2104	0.2628	1.4729	0.4671	0.0563	0.2492
56	0.0687	0.3163	-0.2333	0.4239	0.1250	0.2622
57	0.1354	0.2794	-0.1375	0.5193	0.0396	0.2796
58	-0.2833	0.2596	0.1792	0.4400	0.3333	0.3657
59	0.2958	0.2240	-0.7167	0.5158	-0.0771	0.3484
60	-0.3896	0.3137	-0.4146	0.5255	0.1687	0.2261
61	-0.7208	0.4005	0.6604	0.3852	0.2063	0.2226
62	0.4596	0.4277	0.5085	0.4786	-0.1574	0.2204
63	-0.2178	0.2377	0.3422	0.6514	0.4311	0.3759
64	0.0188	0.2498	-0.3313	0.3963	0.2313	0.3321
65	0.2708	0.3017	0.2083	0.5603	0.1208	0.2729
66	0.0042	0.2657	-0.4937	0.4633	-0.3833	0.3354
67	0.0917	0.3045	-0.2938	0.3303	0.1646	0.1695
